# Workplace Reintegration Facilitator Training Program for Mental Health Literacy and Workplace Attitudes of Public Safety Personnel: Pre-Post Pilot Cohort Study

**DOI:** 10.2196/34394

**Published:** 2022-04-26

**Authors:** Chelsea Jones, Lorraine Smith-MacDonald, Ashley Pike, Katherine Bright, Suzette Bremault-Phillips

**Affiliations:** 1 Heroes in Mind, Advocacy and Research Consortium Faculty of Rehabilitation Medicine University of Alberta Edmonton, AB Canada; 2 Department of Psychiatry Leiden University Medical Centre Leiden Netherlands; 3 Alberta Health Services Edmonton, AB Canada; 4 Department of Occupational Therapy Faculty of Rehabilitation Medicine University of Alberta Edmonton, AB Canada

**Keywords:** public safety personnel, mental health, return to work, reintegration, first responders

## Abstract

**Background:**

Public safety personnel (PSP) impacted by operational stress injuries can find themselves needing both time off work and support reintegrating back into the workforce. Work reintegration programs have been introduced in PSP organizations to support those who aim to return to work. One such peer-led workplace reintegration program (RP) was created in 2009 by members of the Edmonton Police Service (EPS). The primary goal of the EPSRP is to assist PSP in returning to work as soon as possible following a critical incident, illness, or injury while diminishing the potential for long-term psychological injury. The EPSRP is delivered by peers through 3 interrelated components: (1) the Reintegration Program Facilitator Training (RPFT) Program; (2) a short-term Critical Incident RP; and (3) a long-term RP. There is a dire need for research that incorporates strong study designs to the determine long-term effectiveness of the program on increasing workplace reintegration, improving mental health knowledge, and creating culture change within PSP organizations. Simultaneously, the efficacy, effectiveness, and fidelity of the RPFT in providing the tools, mental health knowledge, and skills the RP peer facilitators will need for the RP must be evaluated.

**Objective:**

The purpose of this quasi-experimental pre-post pilot cohort study is to evaluate the effectiveness of the EPSRPFT course on influencing mental health knowledge and attitudes of RPFT attendees who will be future RP peer facilitators.

**Methods:**

This pre-post cohort study collected data via 2 questionnaires from RPFT participants (N=60) which included the Mental Health Knowledge Survey (MAKS) and the Open Minds Survey of Workplace Attitudes (OMSWA). Descriptive, parametric (sample *t* tests), and nonparametric (Wilcoxon signed rank tests) statistics were used to compare the pre- and post-RPFT results and to analyze results by gender and profession.

**Results:**

Statistically significant changes were observed in pre-post questionnaire scores in the domains of mental health attitudes and knowledge.

**Conclusions:**

Although results are explorative, the RPFT may facilitate positive changes in workplace mental health attitudes and knowledge among PSP. It is hoped these findings will contribute to a broader evidence base that can inform changes to the program, practices, and policies, and inform decision-making regarding the EPSRP.

## Introduction

### Background

Public safety personnel (PSP; eg, correctional workers, dispatchers, firefighters, paramedics, police officers) are at an elevated risk of experiencing occupational stress injuries (OSIs) [1]. OSIs which are more common among PSP are work-related psychological distress, mental illness, and work place injuries that result from exposure to events and tasks that can be unpredictable, traumatic, and high risk [2]. A recent study surveying 5813 PSP in Canada found that 36.7% of municipal police, 34.1% of firefighters, 50.2% of Royal Canadian Mounted Police (RCMP), and 49.1% of paramedical staff screened positive for a mental health condition, such as posttraumatic stress disorder (PTSD), depression, anxiety, or substance abuse [3]. These prevalence rates are similar to those in other nations where there are elevated rates of mental health conditions and suicidality among their PSP when compared to the civilian public [4-8]. In an Australian study, 1 in 3 emergency services personnel were reported to experience high or very high psychological distress [7]. In the United States, it is estimated that 30% of first responders develop mental health conditions compared with 20% in the general population [8].

High rates of OSIs can lead to elevated levels of work absenteeism, which risks compromising the level of service that PSP organizations can provide in their communities and increasing the burden on health care and workers’ compensation. OSIs interfere with daily functioning in social, work, or family activities and can leave PSP in need of time off work or unable to return to work in the short- or long-term [9]. These conditions have the potential to impact an individual’s quality of life and can result in decreased community integration, increased social isolation, and greater difficulty forming and maintaining meaningful relationships [10,11]. OSIs such as PTSD can lead to issues with self-care, health, and sleep, all of which can negatively affect success in productive roles such as employment [12]. Productivity-related occupational performance issues attributed to OSIs can cause difficulty with returning to and maintaining work [12].

Facilitating return to work among PSP who have experienced posttraumatic stress injuries and OSIs is a primary concern for PSP, their families, and their organizations. Workplace reintegration interventions offered in clinical health or workers’ compensation settings to address posttraumatic stress injuries and OSIs can be effective in reducing psychological distress and improving daily function. They may have a limited ability, however, to help PSP constructively engage with real-world occupational stressors given that they are offered outside of the PSP service environment [13]. As a result, peer-support interventions and programs offered as an adjunct to clinical evidence-based interventions have become more common within military and PSP organizations.

Peer-support relationships—supportive relationships between people who have a common lived experience—may both enable PSP to take a first step toward recovery and assist individuals throughout the rehabilitation process [14]. Research has illustrated that many PSP feel more comfortable and are more likely to share psychological health challenges with their peers than with a health care professional [15,16]. Commonly shared PSP peer experiences may relate to their career paths and roles as well as mental health challenges or illnesses [14].

Peer-support programs can differ in their area of focus, delivery format and context, and aims. Some programs are associated with critical-incident stress debriefing, critical-incident stress management, peer support, psychological first aid, and trauma risk management [17]. Such programs are delivered in community, clinical, and workplace contexts in both group and one-to-one formats which can contribute to increased accessibility for PSP [14]. Peer-support programs may also be introduced prior to, during, or upon conclusion of clinical interventions and a person’s return to work, and may be the first step a person takes toward recovery. They may also bridge care gaps when geography or organizational security requirements are barriers to care (eg, while clinicians are unable to access PSP environments that require specialized training, certification, and security clearance). In these circumstances, peers can support the therapeutic process within PSP-specific environments, provide a more nuanced understanding of specific occupational stressors and triggers, and help to support or develop creative occupational-specific coping strategies.

Evidence-based literature to support peer-support initiatives among PSP is scarce but emerging. A small body of literature demonstrates promise for peer-support programs in effectively reducing symptoms of psychological distress and increasing self-efficacy and empowerment in civilian and veteran populations with various mental health diagnoses [18,19]. A recent scoping review focused on collating evidence on the use of early interventions for trauma in workers in organizations such as police, fire and rescue, ambulance, and health professionals where traumatic events are routinely experienced due to the nature of the work. With respect to peer-based or peer-led interventions, the reviewers noted that interventions inclusive of peer-group debriefings led to significant reductions in trauma-related absenteeism, and 25 out of 34 studies that delivered an early intervention in a group format found that peer support had facilitated recovery or made for a better experience [20]. A 2015 Finnish survey found that a majority of police officers endorse peer support as a preferred method of learning about stress, health, and posttraumatic treatment [16]. The current literature does not provide information on the functional implications and workplace reintegration outcomes of peer-support programs, which may include workplace presenteeism, work satisfaction, postinjury job demands, perceived organizational justice, perceived work-life balance, and interpersonal workplace relationships as well as the type, intensity, and frequency of the postinjury work (eg, full or part time, modified duties, change in role). Moreover, while some research exists regarding peer-support programs, there is little research evaluating the efficacy and effectiveness of the training that PSP peer-support facilitators receive prior to engaging as leaders in peer-led initiatives.

In 2009, the Edmonton Police Service (EPS) developed a peer-led work reintegration program (RP) that engages police officers in a step-by-step occupation-specific process in actual policing contexts. The primary goal of the EPSRP is to enable officers to return to work as soon as possible, while diminishing the potential for long-term psychological injury [13]. It accomplishes this by engaging the officer in a step-by-step process that addresses the unique stressors that an officer may experience. The pace, scope, depth, and goals of the program are guided by the individual officer with support from the officer’s clinical team. The EPSRP, which includes relationship building, reintroduction to equipment, skill building, exposure therapy, and street exposures [13], is delivered by peers through 3 interrelated components: (1) a RP Facilitator Training Program (RPFT), (2) a short-term Critical Incident RP, and (3) a long-term RP [13]. The short-term Critical Incident RP is offered to support PSP following critical incidents, such as officer-involved shootings. Goals of the long-term RP are to assist officers who have been off work for an extended period of time to return to the normalcy of work settings by providing support and training that are outside the scope of what they have received from their health care provider (ie, psychologist, clinician, or occupational therapist). This study focuses on the first component, the RPFT, and will be outlined in depth in the Methods section.

Since the RP’s inception, 185 EPS officers and 200 plus Alberta Health Services (AHS) emergency services and RCMP K Division staff in Alberta, Canada, have participated in the RP. In the province of Ontario, the Ottawa Police Service and RCMP O Division are actively using the RP with participants while the London Police Service, Ontario Provincial Police, and the Niagara Police Service are in the process of implementing the RP [21]. Evidence regarding the effectiveness of the EPSRP is evolving. Findings from a 2018 internal study by the EPS indicated a 70% reduction in days lost following introduction of the program [13]. A further analysis conducted by the AHS comparing 2 cohorts of emergency services employees demonstrated promising numbers, with 50% more work-days lost in the cohort without the RP compared to the cohort with access to it [21]. Interest in the EPSRP has been stimulated among various PSP groups as a result of positive anecdotal reports. A qualitative analysis of the RPFT was published in 2021, concluding that while the EPSRP holds promise, it is essential that evidence-based research be used to guide RPFT and RP spread and sustainability [22]. PSP organizations in other Canadian provinces, New Zealand, and the United Kingdom are also at various stages of exploring or implementing the EPSRP.

Although PSP organizations are beginning to integrate peer-supported workplace reintegration programs into their mental health strategies, caution must be used. Despite emerging research, a body of scientific literature supporting the efficacy, safety, and effectiveness of peer-lead workplace RPs is lacking, making conclusive decisions about their implementation and use difficult [23]. High-quality RP effectiveness studies that incorporate stronger study designs, rigor, validity, and reliability are needed. Determination of their long-term effects are also required, together with an assessment of potential risk of harm to the participants and facilitators [23]. Research of peer-supported RPs, as well as the facilitator training programs within the Canadian, provincial, and municipal contexts, are needed to ensure that this approach is safe and beneficial for PSP [23,24].

### Objective

The purpose of this quasi-experimental, pre-post, pilot cohort study is to measure changes in the mental health knowledge and attitudes of PSP attendees of the RPFT based on self-reported outcome measures. This study is unique in that it aims to be the first externally peer-reviewed quantitative research regarding this RP that incorporates an a priori study design and includes considerations of rigor, validity, and reliability. The results of this study may assist with the further development, fidelity, and implementation of the RPFT, with the goal of strengthening the RP and initiating further research to meet the aforementioned need for addressing questions regarding efficacy, effectiveness, and safety.

## Methods

### Study Design

This pre-post, quantitative, quasi-experimental, pilot cohort study was part of a larger mixed methods pilot project which employed a convergent parallel design [25,26]. Concurrent collection of quantitative and qualitative data and triangulation occurred once all data were collected.

### Ethical Approval

This study received ethical approval from the University of Alberta Research Ethics Board (study approval #Pro00089517) and was supported by the EPS.

### Reintegration Program Facilitator Training

The 5-day RPFT course was developed and implemented to prepare PSP peers to deliver the EPSRP to colleagues who sought to reintegrate back into work following an OSI and to increase the spread of the RP across PSP organizations. EPS offers the RPFT program multiple times per year for PSP who are interested in becoming RP facilitators or who are implementing an RP within their organizations. The training includes both psychoeducational and experiential components. Examples of psychoeducational topics include the physiological effects of trauma, basic neuroanatomy and physiology, mental health disorders, specific counseling exercises, and interpersonal skills, such as active listening. Experiential components include hands-on graded activities, such as firearms exposures on the firing range for police, mock interrogations for border patrol personnel, donning of equipment for firefighters, and specific scenarios in an ambulance for paramedics. Participants were required to attend and engage in the 40-hour training prior to being designated a RP peer facilitator within their respective PSP organizations.

### Recruitment and Sampling

Participants (N=60) included Alberta-based PSP (eg, RCMP, police, emergency medical services, fire, sheriffs) and clinicians (psychologists and occupational therapists) working with PSPs who attended the RPFT. The participants voluntarily signed up to attend the 5-day RPFT course and had attained approval to participate from their employer. All RPFT attendees were invited to participate in this research study. Upon registering in the RPFT, attendees were asked by the reintegration coordinator if they were interested in participating in the study. Those interested were enrolled and, prior to commencing the RPFT, provided written and verbal consent.

### Outcome Measures

Questionnaires that were administered pre- and posttraining captured descriptive data (eg, age, gender, role), along with information about participant knowledge, skills and attitudes, mental health literacy (Mental Health Knowledge Survey [MAKS]) [27], work reintegration, mental health stigma, and workplace attitude (Open Minds Survey of Workplace Attitudes [OMSWA]) [28].

The MAKS was developed from the theoretical underpinning that stigma comprises 3 constructs: knowledge (ignorance), attitudes (prejudice), and behavior (discrimination) [27]. The MAKS is a 12-item self-report questionnaire designed to measure mental health literacy and stigma [27]. Items are rated on a 5-point Likert scale measuring level of agreement ranging from 1 (strongly disagree) to 5 (strongly agree). Psychometric data support the internal consistency and test-retest reliability of the measure, and the measure appears sensitive to changes in participant responses based on interventions [27,29]. The OMSWA is a 23-item self-report questionnaire designed to measure mental health stigma and workplace attitudes. Items such as “I would be upset if a coworker with a mental illness always sat next to me at work” are rated on a 5-point Likert scale ranging from 1 (strongly disagree) to 5 (strongly agree) [28]. There does exist psychometric data to support the internal consistency of this outcome measure, and it is commonly used by the Mental Health Commission of Canada [29].

A pre-post mental health knowledge questionnaire developed specifically for the EPSRPFT was also trialed based on the RPFT curriculum goals and objectives (Multimedia Appendix 1). This questionnaire employed a 5-point Likert scale ranging from 1 (not at all true) to 5 (very true) for 2 sections: (1) Understanding of Concepts, “I have an excellent understanding of ….” and (2) Skills, “I have excellent skills to ….” A third section required the respondent to rate their level of agreement from 1 (strongly disagree) to 5 (strongly agree) regarding statements related to workplace mental health knowledge, stigma, and attitudes.

### Data Collection

After providing written and verbal consent, and prior to commencing the RPFT, participants were provided a participant number by the lead researcher (CJ) along with a blank envelope containing the precourse surveys. This allowed for participants to be reassured both that their employers were unaware of who participated in the study and their responses and that the study was being conducted externally by independent researchers. Participants were asked to complete the outcome measures and immediately return the envelope to the research team. This process was also completed at the conclusion of the RPFT. Data were then manually entered into SPSS software (IBM Corp) by the research team for analysis.

### Data Analysis

Demographics of the sample were calculated for each of the measures as were total scores for each dependent variable pre- and post-RPFT. Quantitative data were analyzed using SPSS software with sample *t* tests and Wilcoxon signed rank tests. Two-level multilevel modeling analyses were conducted for each repeated measure analysis in the study. Time, as a fixed variable, was coded into 2 time points: (1) baseline and (2) postintervention. Each model included 1 of the dependent variables (ie, MAKS and OMSWA) at each time point (level 1) nested within participants (level 2). Baseline differences in scores among individuals were accounted for by including both a fixed and random intercept in the model. Each final model included the fixed effect of time as the primary predictor variable. All models were computed using the maximum likelihood estimation. All hypothesis testing was conducted using 1-tailed tests at an α level of .05. Cohen *d* effect sizes were computed for all models by standardizing each outcome measure and rerunning the resulting *z* scores in each model. All models were bootstrapped to generate robust probability values and corresponding CIs. A sample of n=30 or higher was required for a power of 0.8, and an attrition rate of 20% was predicted.

## Results

The demographics of the PSP sample are displayed in Table 1. The sample was largely composed of men (n=44), which is common in PSP professions. PSP in policing professions, including municipal policing (n=14) and RCMP (n=12), were the most common among the sample. Of the participants, 32 reported already having an established RP within their home organization.

Statistically significant changes in preintervention to postintervention scores were noted for the overall sample with the MAKS (preintervention: mean 48.70, SD 3.688; postintervention: mean 50.70, SD 3.452; *t*_50_= –3.373; *P*=.001) and are displayed in Table 2. The OMSWA also showed statistically significant preintervention (mean 38.01, SD 9.830) and postintervention (mean 33.18, SD 7.362; *t*_49_=3.692; *P*=.001) changes. This would indicate that among the entire PSP sample, mental health knowledge, literacy, and workplace attitudes toward mental health increased while mental health stigma decreased. The sample was further broken down to analyze the significance of changes on the MAKS and OMSWA by gender and profession (Tables 3 to 6).

The scores of participants who identified as women and completed both pre- and postoutcome measures (n=15) were compared on the pre- and post-MAKS using the Wilcoxon signed rank test (Table 3). Women’s participant scores for posttraining (meaning 20.07, SD 1.32) were lower than those for pretraining (mean 20.93, SD 1.02). The Wilcoxon signed ranks test indicated that the median posttraining ranks on the MAKS were not statistically significantly lower than the pretraining ranks on the MAKS (*z*= –1.67; *P*=.95).

The women’s scores were compared on the OMSWA pre– and post–work reintegration training. The women’s scores posttraining (mean 31.3, SD 1.75) were lower than those pretraining (mean 36.4, SD 2.68). However, the Wilcoxon signed rank test indicated that the median posttraining ranks on the OMSWA were not statistically significantly lower than the pretraining ranks on the OMSWA (*z*= –1.50; *P*=.13).

The scores of those participants who identified as men and completed both pre- and postoutcome measures (n=35) were compared on the MAKS before and after use of the paired sample *t* test (Table 4). The men’s scores posttraining (mean 21.17, SD 0.82) were lower than those pretraining (mean 23.57, SD 0.82). This improvement was statistically significant (*t*_34_= 4.09; *P*<.001). The men’s scores were compared on the OMSWA pre– and post–work reintegration training. Men’s participant scores posttraining (mean 34.37, SD 1.24) were lower than those pretraining (mean 41.06, SD 1.69). This improvement was statistically significant (*t*_35_=3.11; *P*=.004).

Municipal police (n=14) were the most likely to demonstrate a change in the pre-post OMSWA (*z*=–1.97; *P*=.049), demonstrating improvements in workplace attitudes after the RPFT (Table 5). The RCMP (n=12) showed the most statistically significant changes on the MAKS (*z*=–2.37; *P*=.02; median score pretraining 28; median score post training 17.5), which demonstrated an increase in mental health knowledge (Table 6). All other pre-post comparisons of the MAKS and OMSWA for specific PSP professions were nonsignificant.

**Table 1:** Demographic characteristics of the participants (N=60).

Profession		Statistic, n (%)
**Municipal police**		14 (23)
	Men	11 (18)
	Women	3 (5)
**RCMP^a^**		12 (20)
	Men	12 (20)
	Women	0 (0)
**EMS^b^/paramedical**		9 (15)
	Men	4 (7)
	Women	5 (8)
**Sheriff or peace officer**		9 (15)
	Men	7 (12)
	Women	2 (3)
**Firefighter**		6 (10)
	Men	4 (7)
	Women	2 (3)
**Other**		7 (12)
	Men	5 (8)
	Women	2 (3)
**Clinician**		3 (5)
	Men	1 (2)
	Women	2 (3)
**Total**		60 (100)
	Men	44 (73)
	Women	16 (27)

^a^RCMP: Royal Canadian Mounted Police.

^b^EMS: emergeny medical services.

**Table 2:** Overall outcome measure scores.

Outcome measure		Statistic (N=57)		
		Mean score (SD)	*t* value	*P* value
**OMSWA^a^**				
	Pre	38.01 (9.83)	N/A^b^	N/A
	Post	33.18 (7.362)	3.692	.001
**MAKS^c^**				
	Pre	48.7 (3.688)	N/A	N/A
	Post	50.7 (3.452)	–3.373	.001

^a^OMSWA: Open Minds Survey of Workplace Attitudes.

^b^N/A: not applicable.

^c^MAKS: Mental Health Knowledge Survey.

**Table 3:** Pre-post outcome measure scores by gender: women.

Outcome measure		Statistic (N=15)		
		Mean score (SD)	*t* value	*P* value
**OMSWA^a^**				
	Pre	36.4 (2.68)	N/A^b^	N/A
	Post	31.33 (1.75)	–1.50	.13
**MAKS^c^**				
	Pre	20.93 (1.02)	N/A	N/A
	Post	20.93 (1.32)	–1.67	0.95

^a^OMSWA: Open Minds Survey of Workplace Attitudes.

^b^N/A: not applicable.

^c^MAKS: Mental Health Knowledge Survey.

**Table 4:** Pre-post outcome measure scores by gender: men.

Outcome measure		Statistic (N=35)		
		Mean score (SD)	*t* value	*P* value
**OMSWA^a^**				
	Pre	41.06 (1.69)	N/A^b^	N/A
	Post	34.37 (1.24)	3.11	.004
**MAKS^c^**				
	Pre	23.57 (0.82)	N/A	N/A
	Post	21.17 (0.82)	4.90	<.001

^a^OMSWA: Open Minds Survey of Workplace Attitudes.

^b^N/A: not applicable.

^c^MAKS: Mental Health Knowledge Survey.

**Table 5:** Pre-post outcome measure scores for municipal police.

Outcome measure		Statistic (N=12)		
		Mean score (SD)	*t* value	*P* value
**OMSWA^a^**				
	Pre	40.00 (11.19)	N/A^b^	N/A
	Post	35.00 (7.90)	–1.97	.049
**MAKS^c^**				
	Pre	23.21 (5.29)	N/A	N/A
	Post	21.85 (4.05)	–1.33	.18

^a^OMSWA: Open Minds Survey of Workplace Attitudes.

^b^N/A: not applicable.

^c^MAKS: Mental Health Knowledge Survey.

**Table 6:** Pre-post outcome measure scores for Royal Canadian Mounted Police (RCMP).

Outcome measure		Statistic (N=10)		
		Mean score (SD)	*t* value	*P* value
**OMSWA^a^**				
	Pre	38.50 (10.91)	N/A^b^	N/A
	Post	33.63 (6.37)	–1.54	.12
**MAKS^c^**				
	Pre	27.18 (6.78)	N/A	N/A
	Post	20.25 (6.96)	–2.37	.02

^a^OMSWA: Open Minds Survey of Workplace Attitudes.

^b^N/A: not applicable.

^c^MAKS: Mental Health Knowledge Survey.

## Discussion

The purpose of this quasi-experimental, pre-post, pilot cohort study was to evaluate the effectiveness of the EPSRPFT course on influencing mental health knowledge and attitudes of RPFT attendees. The statistical results of the outcome measures, notably the MAKS, OMSWA, and EPSRPFT-specific questionnaire, indicated that the 5-day, 40-hour course likely facilitated increased knowledge of mental health and improved workplace attitudes amongst the participants. The amount of change of the overall scores was statistically significant. Improving mental health knowledge may facilitate a positive impact on stigma, facilitate help seeking, and contribute to a greater proportion of PSP with mental illness engaging in medical treatment [27-30]. It should be noted, however, that knowledge alone is not enough for behavioral change and that additional intrinsic and extrinsic factors, such as cultural awareness, life experiences, the environment, and the responses of others, will also affect whether this knowledge and awareness may influence behavioral change [31,32]. If the EPSRPFT was successful at influencing and facilitating positive change in the level of mental health knowledge, awareness, and skills while improving workplace attitudes in 5 days, this may carry over into the PSP workplace culture and organizations.

Change in scores pre-post training suggests potential gender, profession, organization, and individual differences. Data analysis found that men were more likely to see a larger pre-post change in scores as were municipal police and RCMP. It is hypothesized that the smaller number of women and the smaller subsamples being analyzed for differences between professions might have reduced the sensitivity of the statistical analysis within this study. Gender differences, however, may be important; despite being speculative, the current literature demonstrates that in general, women have greater mental health knowledge or comfort expressing that knowledge to others than do men [33,34]. The lack of change in scores, therefore, does not necessarily indicate that the RPFT program is not meeting its objectives. Rather, it may be indicative that some PSP professions, organizations, and individual PSP had more baseline mental health knowledge upon entering the RPFT and, therefore, did not have as much threshold for change.

Participants appeared to be enrolled in the RPFT for differing reasons. Notably, 53% (32/60) of the sample were from PSP organizations that had already established RPs. It is possible that some of the other participants were enrolled in the RPFT for their own personal growth and learning and did not intend to participate as RP facilitators posttraining. As previously stated, improving mental health knowledge and attitudes can positively influence workplace culture. That being said, it may also be beneficial to reserve the RPFT for those intending to become RP facilitators, with separate learning opportunities available for those participating in the RPFT for other reasons. It is also possible that some of these participants had plans to return to their respective organizations to initiate an RP where one did not previously exist. It may be advantageous to provide information and guidance to this group on how to take steps to implement such a program.

The results of this study should be interpreted with caution, as there were a number of limitations. The data collected in this study were from a single RPFT course specific to a workplace RP without a control group. The specificity of the program and sample limits the generalizability and comparison of the findings. It should also be noted that, although unlikely within the short time period of 5 days, it is possible the observed changes in the PSP attendees mental health knowledge and attitudes could be attributed to other unknown confounding variables aside from the RPFT. A further limitation relates to barriers to open discussion among participants that may arise due to ranks and roles associated with the hierarchical nature of PSP organizations. Stigma surrounding issues regarding mental health, reintegration, PSP culture (norms of hegemonic masculinity, authoritarianism, and emotional control), and organizational culture and policies may also hinder PSP’s confidence in verbally sharing ideas and answering questions. Regarding the sample size, the subsamples had a low number of participants, which might have reduced the sensitivity of the statistical analysis for a given PSP and gender. This is a constant challenge within PSP organizations that can and has been mitigated but is unlikely to be eliminated within the research of this population. As with any study with self-report measures, there are a myriad of issues with self-reporting regarding memory and social desirability biases in reporting. Furthermore, the RPFT-specific questionnaire has yet to be validated. Validation of the questionnaire, which will require more studies with additional participants, may be an asset to the RP in its evaluation as a measure of its effectiveness. Finally, although this study would have benefited from a follow-up 3 and 6 months post-RPFT, the COVID-19 pandemic prevented this from being possible. Despite these limitations and barriers, the overall RP program is experiencing international spread without peer-reviewed research supporting it. It is important to have a pragmatic approach in capturing the RP, as it is currently being deployed in real time to generate preliminary research that uses an evidence-based approach that can be applied within the given context.

More research is needed to evaluate the effectiveness, efficacy, and safety of peer-led workplace RPs and their associated facilitator training programs. As emphasized in the Blue Paper and other research on PSP peer-support initiatives, research conducted by external stakeholders could lead to the evidence-based validation of programs such as the peer-led EPSRP and RPFT program for PSP [23,24,35]. Study of organizational and cultural impact, cost-benefit, implementation drivers and processes, and knowledge mobilization strategies is also warranted. Specifically, studies which employ larger samples would allow for more sensitivity to detecting change in pre-post scores. It is critical that future studies look at the long-term impact of the RPFT and whether mental health competencies, knowledge, and attitudes are maintained over time. As this research expands, other populations at elevated risk of OSIs, such as military, veteran, and health care professionals, could also be included to examine the overall impact of peer-supported workplace reintegration initiatives. Favorable research findings would potentially pave the way for more widespread program adoption and integration that ensures risk management strategies and the maintenance of program fidelity [24]. Use of effective implementation science approaches would best facilitate sustainable spread and scale, enabling more PSP with OSIs to be supported in targeting recovery and return to work. Additionally, the pre-post EPSRPFT questionnaire would benefit from further validation studies to establish parametric data. It may be appropriate to use in future evaluations of other courses related to mental health knowledge and training.

The EPSRP is designed to assist PSP in workplace reintegration after a critical incident or long-term absence from the workplace due to mental or physical health conditions. Evidence-based, curriculum-driven training within programs such as EPSRP may increase return-to-work success among PSP. This pilot study demonstrated preliminary evidence that a 5-day RPFT may contribute to improved mental health knowledge and workplace mental health attitudes among PSP. These newly minted RP facilitators will take this information, perspective, and knowledge from the RPFT into their workplace where they will aim to assist their peers with reintegrating back into the PSP work environment. As a foundational objective evaluation of the RPFT conducted by arms-length researchers, the study responds to the imperative detailed both in the Blue Paper [23] and other publications regarding PSP peer-support programs. It is hoped these findings will contribute to a broader evidence base that can inform changes to the program, practices, and policies, and inform decision-making regarding the EPSRP.

## Appendix

Multimedia Appendix 1 Reintegration Program Facilitator Training (RPFT) Specific Questionnaire.

## Abbreviations

AHS: Alberta Health Services
EPS: Edmonton Police Service
MAKS: Mental Health Knowledge Survey
OMSWA: Open Minds Survey of Workplace Attitudes
OSI: occupational stress injuries
PSP: Public Safety Personnel
PTSD: posttraumatic stress disorder
RP: reintegration program
RPFT: Reintegration Program Facilitator Training
