# Supplementary material for: Workplace Reintegration Facilitator Training Program for Mental Health Literacy and Workplace Attitudes of Public Safety Personnel: Pre-Post Pilot Cohort Study
Source: JMIR Form Res. 2022 Apr 26;6(4):e34394. doi: 10.2196/34394 (PMC9092236; doi:10.2196/34394)
Supplement: Multimedia Appendix 1 [file formative_v6i4e34394_app1.docx]

Reintegration Program Facilitator Training (RPFT) Specific Questionnaire

| **1. I have an excellent understanding of:** |
| --- |
| - Workplace reintegration - Occupational Stress Injury (OSI) - The purpose of the short-term reintegration program - Whom the short-term reintegration program is designed for - Short-term reintegration process - The physiological effects of a critical incident - The psychological effects of a critical incident - Interaction Training - Post-Traumatic Stress Disorder (PTSD) - Symptoms of PTSD - Effect of PTSD on the Brain - The purpose of the long-term reintegration program - Whom the long-term reintegration program is designed for - Long-term reintegration process - Resilience - How to build trust with participants of a workplace reintegration program - The unique strengths and challenges of various public safety personnel - The role of healthcare clinicians in mental health - Exposure Therapy - Psychotherapeutic techniques for those affected by trauma - Subjective units of distress scale (SUDS) - Mental Health First Aid - Empathy - Post-Traumatic growth |
| **2. I have excellent skills to:** |
| - Support colleagues in workplace reintegration - Identify strengths and challenges in reintegration processes - Build trust with participants in the reintegration process - Communicate with participants in the reintegration process - Address Mental Health Stigma in the workplace |
| **3. Please indicate your level of agreement with the following statements:** |
| - I understand how mental health problems present in the workplace. - I plan to seek help for my own mental health problems if needed. - When I am concerned, I ask my colleagues how they are doing. - I think about mental health issues as freely as physical health issues. - I understand management practices that promote the mental well-being of all. - I will be a better leader now that I have completed the EPS Reintegration Trainer Training. - I will have better mental health now that I have completed the EPS Reintegration Trainer Training. - I will use the skills I have learned in the EPS Reintegration Trainer Training to be part of a peer-supported workplace reintegration program within my organization. |

RPFT Specific Questionnaire. Section 1 and 2: 5-point Likert scale ranging from 1 (not at all true) to 5 (very true). Section 3: 5-point Likert scale ranging 1 (strongly disagree) to 5 (strongly agree).
